# Challenges of young scientists at the cutting-edge of genotoxicity research: the open symposium of the Japanese Environmental Mutagen Society (JEMS), 2018

**DOI:** 10.1186/s41021-018-0111-6

**Published:** 2018-11-02

**Authors:** Manabu Yasui, Shigeharu Muto, Akira Sassa

**Affiliations:** 1 Division of Genetics and Mutagenesis, National Institute of Health Sciences, 3-25-26 Tono-machi, Kawasaki-ku, Kawasaki, Kanagawa 210-9501 Japan; 2 Safety Research Laboratories, Mitsubishi Tanabe Pharma Corporation, 2-2-50, Kawagishi, Toda-shi, Saitama, 335-8505 Japan; 3 Department of Biology, Graduate School of Science, Chiba University, 1-33 Yayoi-cho, Inage-ku, Chiba, 263-8522 Japan

**Keywords:** Genotoxicity test, DNA damage, Mutagenesis, Carcinogenesis, Environmental mutagen, Cancer

## Abstract

The Open Symposium of the Japanese Environmental Mutagen Society (JEMS) entitled “Challenges of Young Scientists at the Cutting-edge of Genotoxicity Research” was held in the Main Conference Room of the Foundation for Promotion of Cancer Research, Tokyo, on June 9th, 2018. This year, the symposium aimed to provide an opportunity to highlight the cutting-edge research activities of young scientists who are continuing to expand frontiers of the fields of environmental mutagenesis and genetic toxicology; it also aimed to inform JEMS activities to the participants. Through this report, the organizers present a summary of the symposium.

## Background

The Open Symposium of the Japanese Environmental Mutagen Society (JEMS) is annually organized to present JEMS’ research in the fields of genetic toxicology and environmental mutagenesis to the public, and its proceedings are summarized in meeting reports [1–4]. Last year, the symposium was entitled “Research on Environmental Mutagenesis from Young Scientists” and was organized by Dr. Kenichi Masumura and Dr. Shuichi Masuda, particularly for young scientists, who were provided an opportunity to present their research to a broad audience. In 2018, JEMS has still a lot of talented young researchers with a good balance of representation from the industrial, governmental, and academic sectors, all connected by their research in genetic toxicology and environmental mutagenesis. Consistent with the last year, the organizers Manabu Yasui, Shigeharu Muto, and Akira Sassa, who are also young researchers, designed a JEMS Open Symposium of, by, and for young scientists to other scientists and to the public. The 2018 Open Symposium entitled “Challenges of Young Scientists at the Cutting-edge of Genotoxicity Research” was held in the Main Conference Room of the Foundation for Promotion of Cancer Research, Tokyo, on June 9th. The aim of the symposium was to provide an opportunity to present cutting-edge research activities of young scientists who were continuing to challenge in the notable the fields of environmental mutagenesis and genetic toxicology. The organizers defined the term “cutting-edge” as work that not only demonstrates the highest level of research using state-of-the-art apparatus and techniques but also pioneers an unexplored research field. Eight young scientists were selected from among the JEMS members to present their work at the symposium. Through this report, the organizers present a summary of the event.

## Symposium program

Masamitsu Honma (President, JEMS: National Institute of Health Sciences): Inaugural speech.

Manabu Yasui (National Institute of Health Sciences): Introduction.

Session 1 (Chairs: Shigeharu Muto and Masashi Sekimoto).

Miyuki Shigano (LSI Medience Corporation): Liver micronucleus assays using a preparation method from formalin-fixed tissues.

Emiko Okada (Yakult Honsha Co., Ltd.): Development of in vivo gastrointestinal tract micronucleus assay.

Tatsuya Kato (Mitsubishi Tanabe Pharm Corporation): Genotoxicity assessment based on mechanism of action in pharmaceutical development.

Session 2 (Chairs: Manabu Yasui and Daisuke Nakajima).

Katsuyoshi Horibata (National Institute of Health Sciences): Performance of *Pig-a/ PIG-A* gene mutation assay as in vivo genotoxicity tests including human samples.

Akira Sassa (Chiba University): DNA and RNA–genomic instability caused by a slight structural difference.

Ayumi Yamamoto (National Institute of Technology, Hachinohe College): Environmental mutagen study and education from the viewpoint of the food field.

Session 3 (Chairs: Akira Sassa and Kei-ichi Sugiyama).

Masako Oka (Fukuoka Dental College): Establishment of human iPS cells with mitochondrial complex II deficiency as cancer models.

Wataru Sakai (Kobe University): A new insight into the pathogenesis of Fanconi anemia: origin of endogenous DNA damage.

Shigeharu Muto (Mitsubishi Tanabe Pharm Corporation): Concluding speech.

## Meeting report

Ms. Miyuki Shigano presented her research on an improved method for staining hepatocytes present in formalin-fixed liver tissues for micronucleus (MN) assays; this method did not require collagenase treatment. The liver sample used had been fixed with 10% phosphate-buffered formalin approximately 5 years previously, demonstrating that even liver tissues that have been stored for a relatively long duration can be tested using the MN assay. This method not only enables integration of the liver MN assay into general repeated-dose toxicity studies but also allows it to be retrospectively conducted.

Dr. Emiko Okada reported her research on the development of an in vivo rat gastrointestinal (GI) tract MN assay. The GI tract, particularly the stomach, is the first site of contact for test chemicals administered by oral gavage. Dr. Okada also described a collaborative study conducted by the Mammalian Mutagenicity Study group (MMS) that belongs to JEMS to evaluate the suitability of the repeated-dose liver and GI tract MN assays. The stomach MN assay could detect the clastogenicity of three test chemicals, including a stomach-targeted carcinogen. Moreover, additional verification studies using three GI tract-targeted genotoxic carcinogens as well as non-carcinogens found that all carcinogens produced positive results and all non-carcinogens produced negative results, indicating that the GI tract MN assay is useful for evaluating the genotoxicity of orally administered compounds.

Dr. Tatsuya Kato presented his research on mechanism-based genotoxicity risk assessment in pharmaceutical development. His investigation regarding DNA adduct formation by 2,4- and 2,6-diaminotoluene in rat liver and *Salmonella typhimurium* using DNA adductome analysis revealed that differences in metabolism might cause differences in the amount and the structure of DNA adducts. Moreover, Dr. Kato described the development of a fundamental novel method to elucidate the relationship between functional inhibition and genotoxicity using an siRNA approach, which is important because inhibition of proteins playing important roles in cellular function is a possible genotoxic mechanism. The approach is useful to elucidate the mechanism of action of test compounds, and to discuss the validity of pharmacological target itself (on- or off-target for genotoxicity).

Dr. Katsuyoshi Horibata explained how the *Pig-a* gene mutation assay (*Pig-a* assay), a novel in vivo genotoxicity test, can be used to analyze accumulated and quantitative genotoxicities and how it is advantageous over conventional methodologies like MN tests. Dr. Horibata verified the superiority and usefulness (it can be detected by short term test) of PIGRET assay, developed in Japan during collaborative research at the JEMS/ MMS Study Group, and succeeded in making international contributions to be OECD guideline application. In addition, he established a human *PIG-A* assay to monitor human genotoxicity and evaluated genotoxicity in human blood samples obtained from subjects undergoing chemotherapy and radiotherapy. He found strong genotoxicity signals in 2 out of 27 patients receiving chemotherapy, indicating that the genetic toxicity test may be useful in humans.

Dr. Akira Sassa explained how RNA precursors, i.e., ribonucleotides are sometimes misincorporated during DNA replication. In the absence of ribonucleotide excision repair (RER), ribonucleotides accumulate in the genome, resulting in various abnormalities, such as DNA replication delay, DNA damage response activation, and epigenetic dysfunction. He also explained that defects in RER are associated with the Aicardi–Goutières syndrome, a serious human autoimmune disease. He particularly focused on the “mutagenic potential” of a ribonucleotide incorporated into DNA because even a single ribonucleotide can cause serious DNA mutations, such as large deletions, which can be suppressed by particular repair pathways other than RER. His study suggests new possible mechanisms to protect cells against the deleterious effects of ribonucleotide misincorporation into DNA.

Dr. Ayumi Yamamoto explained how decreasing the prevalence of cancer and lifestyle-related diseases is crucial for extending healthy life expectancy, reducing medical costs, and facilitating more comprehensive life planning. Thus, protecting genomic DNA from various types of stress is of key importance for the prevention of the above mentioned pathologies. He suggested that DNA-protective agents, such as those found in foods that inhibit DNA damage and gene mutation, should be called “genome defenders;” these were previously called “antimutagenic substances,” but such a term is unfamiliar to consumers. Further, he elaborated on how environmental mutagen studies in foods are a topic of deep interest in his laboratory. He also explained the potential for blackcurrant as a food-based genome defender.

Dr. Sugako Oka reported her research on the establishment of a new experimental model using human iPS cells to elucidate the role of oxidative stress in cancer development. Through this model, reactive oxygen species (ROS) levels can be elevated by expressing a mutant form of the mitochondrial complex II subunit “SDHC,” which is strictly regulated by the Tet-on ProteoTuner system. In addition, ROS levels can be suppressed at any given time via the expression of the antioxidant enzyme “catalase.” The abovementioned model can be used to identify the signals that initiate carcinogenesis as well as to elucidate the biological impact of ROS by analyzing the responses of iPS cells (e.g., differentiation and programed cell death).

Dr. Wataru Sakai described his research regarding a new possible mechanism for the suppression or repair of “metabolic DNA lesions,” which he defined as the DNA lesions that can occur via metabolic reactions in vivo. He identified a factor involved in lipid aldehyde metabolism as potentially interacting with the Fanconi anemia (FA) protein “FANCD2.” He also investigated the direct interaction of that factor with FANCD2 and its involvement in DNA damage response. This study provides a new insight into the role of the FA pathway in preserving genome integrity.

Approximately 104 participants attended the symposium, and a questionnaire survey revealed that 32% of the attendees were not JEMS members. As the organizers, we would like to thank to everyone who attended this symposium.

## Abbreviations

FA: Fanconi anemia
GI: Gastrointestinal
JEMS: Japanese Environmental Mutagen Society
MMS: Mammalian Mutagenicity Study group
MN: Micronucleus
RER: Ribonucleotide excision repair
ROS: Reactive oxygen species
